# Epidemiological, clinical and virological characteristics of four cases of monkeypox support transmission through sexual contact, Italy, May 2022

**DOI:** 10.2807/1560-7917.ES.2022.27.22.2200421

**Published:** 2022-06-02

**Authors:** Andrea Antinori, Valentina Mazzotta, Serena Vita, Fabrizio Carletti, Danilo Tacconi, Laura Emma Lapini, Alessandra D’Abramo, Stefania Cicalini, Daniele Lapa, Silvia Pittalis, Vincenzo Puro, Marco Rivano Capparuccia, Emanuela Giombini, Cesare Ernesto Maria Gruber, Anna Rosa Garbuglia, Alessandra Marani, Francesco Vairo, Enrico Girardi, Francesco Vaia, Emanuele Nicastri, the INMI Monkeypox Group, INMI Monkeypox Group, Alessandro Agresta, Francesco Baldini, Tommaso Ascoli Bartoli, Alessia Beccacece, Rita Bellagamba, Aurora Bettini, Nazario Bevilacqua, Marta Camici, Francesca Colavita, Angela Corpolongo, Gabriella De Carli, Federico De Zottis, Francesca Faraglia, Massimo Francalancia, Concetta Maria Fusco, Roberta Gagliardini, Saba Gebremeskel, Maria Letizia Giancola, Giulia Gramigna, Elisabetta Grilli, Susanna Grisetti, Simone Lanini, Gaetano Maffongelli, Andrea Mariano, Ilaria Mastrorosa, Giulia Matusali, Silvia Meschi, Claudia Minosse, Martina Moccione, Annalisa Mondi, Vanessa Mondillo, Nicoletta Orchi, Sandrine Ottou, Carmela Pinnetti, Silvia Rosati, Martina Rueca, Laura Scorzolini, Eliana Specchiarello, Alessandra Vergori.

**Affiliations:** 1 National Institute for Infectious Diseases ‘Lazzaro Spallanzani’ (IRCCS), Rome, Italy; 2 Department of Specialised and Internal Medicine, Infectious Diseases Unit, San Donato Hospital, Arezzo, Italy; 3 Infectious and Tropical Disease Unit, AOU Policlinico Umberto I, Rome, Italy; 4 The members of the group are listed under Collaborators

**Keywords:** monkeypox, MSM, emerging infectious diseases, west African clade, seminal fluid

## Abstract

Since May 2022, an outbreak of monkeypox has been ongoing in non-endemic countries. We report four cases in Italy in young adult men reporting condomless sexual intercourse. The patients are in good clinical condition with no need for specific antiviral drugs. Biological samples from seminal fluid were positive for monkeypox viral DNA. For many other viruses found in semen there is no evidence of sexual transmission. The possibility of sexual transmission of monkeypox virus needs to be investigated.

Human monkeypox virus (MPXV) is a double-stranded DNA virus of the *Orthopoxvirus* genus of the *Poxviridae* family. Two genetic MPXV clades have been characterised: West African and Central African. Outside of Africa, the first cases of monkeypox were reported in 2003 in the United States (US) [1] when an outbreak occurred following the importation of rodents from Africa. Over the past five decades, monkeypox outbreaks have been reported in 10 African countries and four countries outside of Africa [2]. To date, in the ongoing outbreak in 2022, 118 cases of monkeypox have been reported in non-endemic countries [3]. We report four monkeypox cases observed in Italy between 17 and 22 May 2022.

## Case description

All four monkeypox patients were young adult men who have sex with men (MSM). Two of them (Patients 1 and 3) had an HIV infection and received effective antiretroviral therapy (ART); the other two (Patients 2 and 4) were on antiretroviral pre-exposure prophylaxis (PreP).

All patients travelled in the first 2 weeks of May 2022: three patients participated in a mass gathering event in Gran Canary island (GCI) and one travelled for sex work. During the travel, they had condomless sexual intercourse with different male partners.

All four patients had a history of sexually transmitted infections (STIs) (Table 1).

**Table 1:** Clinical and epidemiological characteristics, monkeypox patients, Italy, May 2022 (n = 4)

Characteristics	Patient 1	Patient 2	Patient 3	Patient 4
Sex	Male	Male	Male	Male
Age (years)	30s	30s	30s	30s
Sexual behaviour	MSM	MSM	MSM	MSM
Previous STIs	Hepatitis C, syphilis	Syphilis	Syphilis, hepatitis B	Hepatitis A^a^
HIV status	Positive	Negative on PrEP	Positive	Negative on PrEP
Recent sexual exposure	Yes	Yes	Yes	Yes
Systemic symptoms	No	Fever	Fever	Myalgia
Days from systemic symptoms to appearance of lesion	NA	3	3	2
Localisation of skin lesions	Genital, thorax and calf area	Anal, back, legs and foot sole	Anal, head, thorax, legs, arms, hand, and genital area	Genital and pubic area
Evolution of lesions	Asynchronous	Asynchronous	Asynchronous	Asynchronous

MSM: men who have sex with men; NA: not applicable; PrEP: pre-exposure prophylaxis; STI: sexually transmitted infection.

^a ^Acquired as part of the hepatitis A cluster in MSM, in Europe in 2017.

All four monkeypox patients were in good clinical conditions and were admitted to two different hospitals in central Italy, in combined droplet and contact isolation measures plus filter face piece-2 (FFP2) for clinical care management.

The first case was a male in his 30s who referred to our centre after accessing an emergency department where an MPXV infection was suspected. During travel made in mid-May, he had been treated with oral ciprofloxacin and acyclovir and one single dose of benzylpenicillin for the appearance of skin lesions. At admission, multiple asynchronous deep-seated and well-circumscribed lesions with central umbilication were present on the genital area, with inguinal lymphadenopathy; a single lesion was present on the anterior and posterior thorax and on the left calf.

Patient 2 was a male in his 30s, taking daily-PreP. He was admitted for fever and asthenia starting mid-May. Three days later, perianal lesions appeared as raised, itchy papules secreting serous, with concomitant painful inguinal lymphadenopathy. Multiple anal lesions appeared over the next 3 days, followed by few single lesions with different timing on the skin of the back, legs and the sole of one foot.

Patient 3 was a male in his 30s. He was admitted for a 2-day history of fever, followed by the appearance of clustered itchy papular lesions in the anal region and single lesions on head, thorax, legs, arms, hand and penis. He reported smallpox vaccination during childhood, 30 years earlier.

Patient 4 was a male in his 30s, taking event-driven PreP. After a 2-day history of myalgia, vesicular-papular genital lesions appeared, followed by further skin lesions that appeared 6 days later in the suprapubic area and chest.

**Figure 1:**
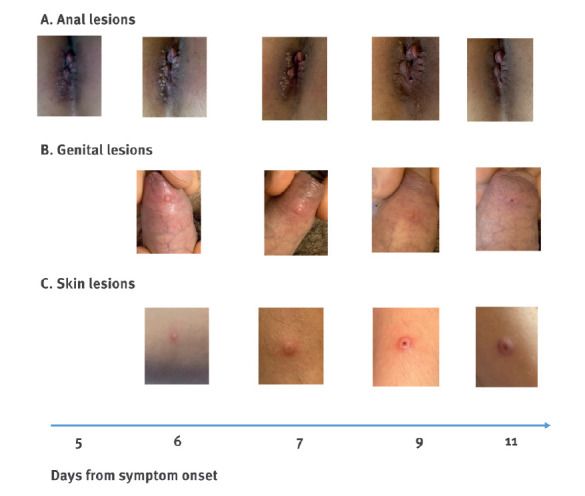
Pictures from 2 patients with the horizontal disease progression from the same patient for each site, monkeypox cases, Italy, May 2022

In all patients, skin lesions progressed with an asynchronous evolution (Figure 1).

Lesions appeared 1–3 days after systemic symptoms, clustered (anal area 1A) or isolated (skin or penis 1B-1C). They typically began as raised, itchy papules secreting serous, with a central umbilication; over days, the central umbilication widened until the lesion opened and the scab formed about 2 weeks after symptom onset.

Samples obtained from skin, genital and anal lesions, serum, plasma, seminal fluid, faeces, and nasopharynx were all positive for MPXV DNA in real-time PCR (Table 2). The patients did not experience any systemic symptoms during clinical observation, and they recovered spontaneously, without any specific antiviral therapy. In Patient 2 only, anti-inflammatory and antihistaminic drugs were used for perianal pain and general itch.

**Table 2:** Timeline of PCR results, monkeypox cases, Italy, May 2022 (n = 4)

	Patient 1		Patient 2			Patient 3				Patient 4
Day after symptom onset	Day 5	Day 9	Day 3	Day 5	Day 9	Day 5	Day 6	Day 8	Day 11	Day 4
Serum	Pos (29.7)	NA	AO	AO	NA	AO	AO	NA	NA	AO
Plasma	Pos (30.2)	NA	AO	AO	NA	NA	AO	NA	NA	AO
Genital or rectal lesions	Pos (15.6)	NA	Pos (17.5)	AO	NA	Pos (15.3)	NA	NA	NA	Pos (14.7)
Nasopharyngeal swab	Pos (27.6)	AO	Pos (30.2)	NA	NA	NA	AO	NA	NA	Pos (30.4)
Skin lesions	NA	NA	Pos (30.4)	AO	NA	Pos (18.2)	Pos (19.4)	NA	NA	Pos (17.6)
Seminal fluid	NA	Pos (30.1)	NA	Pos (29.4)	Pos (43.2)	NA	Pos (29.3)	Pos (27.7)	Neg	NA
Scab	Pos (13.1)	NA	NA	NA	NA	Pos (20.0)	NA	NA	NA	NA
Faeces	NA	NA	Pos (22.6)	NA	NA	NA	Pos (26.1)	NA	NA	NA
Saliva	NA	NA	Pos (27.1)	NA	NA	NA	AO	NA	NA	NA

AO: analysis ongoing; Cq: quantification cycle; NA: not available; neg: no detection of monkeypox DNA; pos: detection of monkeypox DNA.

Cq values are indicated in brackets after positive results.

## Laboratory diagnosis and virus identification

Viral DNA was extracted by Qiamp Viral RNA mini kit (Qiagen), and two real-time PCRs were used to assess the presence of MPXV DNA. Real-Star Orthopoxvirus PCR Kit (Altona Diagnostics GmbH) was used as screening PCR. This method recognises a region common to all Orthopoxviruses without distinction of species. The second PCR (G2R_G assay) published by Li et al. targets the tumour necrosis factor (TNF) receptor gene and was used as confirmatory PCR [4]. The Rotor Gene Q (Qiagen) platform was used to run both assays. We also measured viral quantification cycle (Cq) in positive samples.

## Sequencing, bioinformatic and phylogenetic analysis

We performed Sanger sequencing of the amplicon obtained from a third end point PCR (in-house method), targeting the complete gene for the viral haemagglutinin (HA) to identify which of the two known MPXV clades, the virus in the patient samples belonged to. We obtained the HA gene sequence only for the first three patients. The sequence of the fourth patient is in progress.

For the first case, next generation sequencing was performed on an Ion Torrent GSS5 platform (ThermoFisher), using a metagenomic approach starting from 100 ng extracted DNA. The library was prepared using the Ion Xpress Plus Fragment Library Kit (Thermofisher). After quality filter, the reads were de-novo assembled using SPAdes [5]. Major contigs were selected and aligned with BWA v.0.7.12 [6], to recently published MPXV sequences, and merged in a unique contig. The final length resulted in 190,280 nt, with a mean coverage of 159 reads. At the end, the assembled genome of MPXV-INMI-Pt1 was annotated, transferring the annotation from the sequence MPXV-UK_P1 (MT903343.1 1) with Geneious Primem and included 186 open reading frames. The sequence was submitted on 25 May and released on 26 May on GenBank (Accession number: ON614676.1).

**Figure 2:**
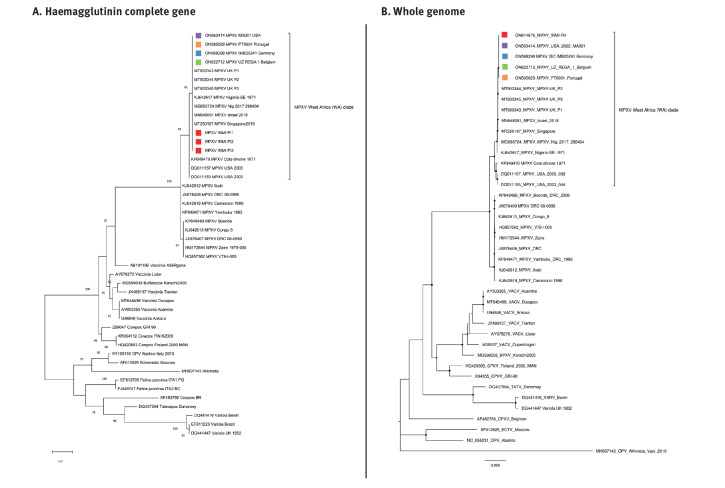
Phylogenetic tree of monkeypox cases, Italy, May 2022 (n = 4)

We performed phylogenetic analyses using INMI sequences (HA and full genome) (Figure 2) and sequences related to the HA gene of different Orthopoxviruses retrieved from GenBank (Belgium, Germany, Portugal and the United States), including MPXV sequences belonging to the two clades West Africa and Central Africa. The phylogenetic trees were built using the maximum likelihood method and evolutionary distances were computed using the Tamura 3-parameter model for single gene tree and Kimura three parameters for the full genome analysis. Bootstraps were generated using 1,000 replicates.

MPXV: monkeypox virus.

The INMI patient sequences described in the text are highlighted with a red square. Other sequences from monkeypox patients from Belgium, Germany, Portugal and the United States are highlighted with coloured squares (Belgium in green, Germany in light blue, Portugal in orange and United States in violet). All sequences highlighted were reported in May 2022. The GenBank accession numbers are indicated in the tree.

Panel A: A total of 46 sequences were included: 43 sequences related to the HA gene of different *Orthopoxviruses* and MPXV sequences belonging to the two clades, West Africa and Central Africa; the remaining three INMI patient sequences were from Patients 1, 2 and 3 described in this manuscript.

Panel B: A total of 40 whole genomes of different *Orthopoxviruses* and MPXV sequences belonging to the two clades West Africa and Central Africa including Patient 1 described in this manuscript.

## Discussion

The clinical description of, to our best knowledge, the first four monkeypox cases detected in Italy warrants closer investigation for several reasons. Firstly, it has been suggested that the MPXV in the currently ongoing outbreak is transmitted from human to human [7], even though this route of transmission has been reported as poorly efficient in previous outbreaks caused by the West African clade [8]. Secondly, the clinical picture seems to be different from the available information in the literature because the skin lesions in our patients were asynchronous, ranging from single or clustered spot to umbilicated papule with progressive central ulceration and, finally, to scabs. Lesions were mostly located in genital and perianal sites. Finally, the individuals affected by monkeypox are mostly men [9]. In endemic areas, most of the cases were observed in men [10], probably related to hunting practices, whereas in the current outbreak, most individuals are MSM, people with multiple sexual partners or people who practice condomless sex.

Sexual behaviour of the cases in the present series and the initial appearance of lesions mostly in the anal and genital areas all suggest that close contact during sexual intercourse was important for virus transmission. The seminal fluid obtained from three monkeypox patients at the time closest (5–7 days) to symptoms onset, was found to be positive for MPXV DNA in all four patients, with a Cq range from 27 to 30. The correlation between Cq value and infectious viral load in MPXV infections is not yet known, but the range of Cq values we measured in semen makes viral isolation unlikely. However, detection of viral DNA in the three seminal fluid samples we analysed, excludes the hypothesis of biological sample contamination. Although these findings cannot be considered definitive evidence of infectivity, they demonstrate viral shedding whose efficiency in terms of transmission cannot be ruled out. It has been observed that it is likely that MPXV can be transmitted through substances of human origin [11,12] and it needs to be emphasised that the Cq values in semen of our patients were in the range of those measured in their nasopharyngeal swabs.

We need to consider that many other viruses causing viraemia can be found in semen [13,14] with no direct evidence of sexual transmission. Indeed, viral seeding in the male reproductive tract can frequently occur in the context of viraemia, as blood–testis barriers are imperfect for viruses, especially in the presence of systemic or local inflammation [15]. Since testes are an immunologically privileged sanctuary site, the virus may persist even though unable to replicate within the reproductive tract.

## Conclusion

The characteristics of these four patients reflect those described in other European countries in the current outbreak. The phylogenetic characteristics of the virus could support the hypothesis of a recent introduction of the West African clade of the MPXV into the community in non-endemic countries. Moreover, the characteristics of the population involved, as well as reported exposure to multiple, condomless sexual contacts, suggest that human-to-human transmission through close physical contact in sexual networks plays a key role in the current outbreak. Further studies are needed to assess presence, persistence and contagiousness of MPXV in different body fluids.
